# Phenological, Physiological, and Ultrastructural Analyses of ‘Green Islands’ on Senescent Leaves of Norway Maple (*Acer platanoides* L.)

**DOI:** 10.3390/plants14060909

**Published:** 2025-03-14

**Authors:** Violetta Katarzyna Macioszek, Kamila Chalamońska, Jakub Oliwa, Aleksandra Maria Staszak, Mirosław Sobczak

**Affiliations:** 1 Laboratory of Plant Physiology, Department of Biology and Plant Ecology, Faculty of Biology, University of Bialystok, 15-245 Bialystok, Poland; 2 Faculty of Agriculture and Ecology, Warsaw University of Life Sciences (SGGW), 02-787 Warsaw, Poland; 3 Institute of Biology and Earth Sciences, University of the National Education Commission, 31-054 Krakow, Poland; jakub.oliwa@gmail.com; 4 Department of Botany, Institute of Biology, Warsaw University of Life Sciences (SGGW), 02-787 Warsaw, Poland

**Keywords:** chlorophyll, ‘green islands’, Norway maple, phenolic compounds, photosynthesis, *Rhytisma* spp. senescence

## Abstract

‘Green island’ symptoms in the form of vivid green, round spots visible on the senescent leaves of many plants and trees are mostly the results of pathogenic colonization by fungi, and the greenish tissue is often dead. Therefore, this study investigates whether green spots observed on senescent Norway maple (*Acer platanoides* L.) leaves were still alive and photosynthetically active. The appearance of ‘green islands’ on the leaves of young Norway maple trees was observed from the autumn of 2019 to 2022 in an urban forest (Bialystok, eastern Poland). However, in the late summer (September) of 2023 and 2024, mostly tar spots caused by the fungus *Rhytisma* spp. on maple leaves could be observed, with only a few leaves having ‘green island’ symptoms. The percentage of ‘green island’ areas on senescent leaves observed during the 4 years (2019–2022) was influenced by a year of sampling (*p* < 0.001). A non-destructive physiological analysis of chlorophyll, flavonoids, and nitrogen balance index (NBI) in leaves revealed that these parameters were significantly lower in ‘green islands’ than in the summer leaves, but higher than in the senescent yellow area of the autumn leaves. In the case of anthocyanins, their level was significantly higher in ‘green islands’ than in yellow areas, although, in the summer leaves, anthocyanins were undetectable. The amount of chlorophyll and most photosynthetic parameters were significantly (*p* < 0.05) reduced in the ‘green islands’ of the senescent leaves compared to the mature green leaves. However, these parameters were significantly higher in the ‘green islands’ than in senescent yellow leaves. Carotenoid content in the ‘green island’ and yellow areas of senescent leaves were at the same level, twice as higher than in summer leaves. Green mature leaves and the ‘green islands’ on senescent leaves had the same structure and anatomy. The main differences concerned the chloroplasts, which were smaller and had less grana and starch grains, but had more plastoglobuli in ‘green island’ cells. The cells building the mesophyll in the yellow area of the leaf deteriorated and their chloroplasts collapsed. Epiphytes were present on the adaxial epidermis surface in all types of samples.

## 1. Introduction

Norway maple (*Acer platanoides* L.) is a tree species from the soapberry family (*Sapindaceae* Juss.). It occurs naturally in Central and Eastern Europe and in the Balkans and Caucasus [1,2]. However, it is considered an invasive species in North America [3,4]. It is the most common of the maple species found in Poland and is characterized by a spreading, umbrella-like crown. *A. platanoides* grows to approx. 18–27 m in height, and the trunk width can reach up to 1.5 m. The trunk is covered with thin bark, red-brown in a young twig, and it darkens and becomes furrowed on old branches [5,6]. Maple stems are straight and short, with numerous perpendicular shoots, covered with opposite leaves. The leaves have five lobes with long, pointed teeth and smooth edges. Their size depends on the age and condition of the plant [7].

Norway maple is widely used as an ornamental, shade, and street tree due to its attractiveness and tolerance to specific urban conditions. It can regenerate vigorously after pruning, so it can be used as a living fence [8]. Due to the production of fine adventitious roots, maple can be planted in mountainous areas, thus protecting the soil against excessive erosion [9] and stabilizes slopes reducing rockfalls [10]. In natural habitats, Norway maple is almost completely free from serious diseases. However, in highly urbanized areas, it may suffer from various diseases caused by a combination of stresses resulting from excessive air and soil pollution [8].

In general, morphological changes in the form of ‘green islands’, i.e., fragments of the surface of leaf lamina retaining a green color on senescent and yellowing tree leaves, are most often associated with the local presence of endophytic microorganisms, mainly fungi and bacteria [11]. Endophytes are microorganisms usually having a beneficial effect on plants or benign parasites and pathogens that do not cause disease symptoms, inhabiting the interior of plant organs for most of their life cycle [12,13]. However, a term “endophyte” rather refers only to the place of their occurrence, and not to their impact on the metabolism and the physiological or health condition of plants, also concerning pathogenic microbes [14]. The employment of genome and metagenome sequencing enabled the identification of some endophyte species and the preliminary determination of the mechanisms of their interaction with various species of infected plants in ‘green islands’ [15]. The phenomenon of ‘green island’ is most often described and observed on plants infected with pathogenic microorganisms [11]. The area around the site of pathogen infection remains green while the surrounding leaf tissues degrade and naturally age.

However, ‘green islands’ formed on senescent and yellowing leaves of trees, leaves of herbaceous plants, and crops infected by various pathogens do not have the same structure. Thus, it enables easy identification of ‘green islands’ with living host cells and pathogens, as well as those in which pathogen cells remain alive, while the cells of the host plant are dead or dying [11,16]. The phenomenon of ‘green islands’ is related to interactions between the plant and biotrophic fungal pathogens, such as powdery mildew or brown rust [17], as well as with some hemibiotrophic fungi, e.g., *Plasmodiophora brassicae* and *Colletotrichum graminicola* [18], or with parasitism of some insects [19]. It has also been observed that ‘green islands’ may appear in places of infection with necrotrophic fungi such as *Pyrenophora teres* or *Alternaria brassicicola* [20,21]. Considering the number of pathosystems in which green islands are induced, it seems unlikely that there is one universal and common mechanism responsible for the formation or retention of photosynthetically active cells in degrading leaves. The appearance of ‘green islands’ on infected and degrading leaves is a beneficial phenomenon for fungal pathogens, as it significantly extends the life of host cells. However, the etiology, origin, structure, functions, and environmental significance of these structures have not yet been fully explained, although the scale of this phenomenon is extensively expanding [11]. Undoubtedly, ‘green islands’ that maintain photosynthesis function in infected areas have less impact on plant health in trees as they lose leaves in autumn than crops. However, this phenomenon can be a visible marker in crop plants’ practical disease diagnosis and management.

‘Green islands’ in *A. platanoides* are visible in autumn when leaves begin to yellow. It has been found that this phenomenon in Norway maples in Europe is most probably induced by a pathogenic fungus *Sawadaea bicornis*. It has at least been recognized as a dominant species in *A. platanoides* ‘green islands’, together with other endophytic fungi and bacteria [15].

The aim of this study was to show the variability of dominant disease symptoms on leaves of Norway maple growing in the urban forest of Bialystok during the past 6 years, with an emphasis on ‘green island’ development. Moreover, investigating naturally occurring green islands might be a valuable model for investigating how pathogens manipulate the host plant metabolism. Therefore, physiological and ultrastructural changes within the ‘green islands’ of senescent leaves of *A. platanoides* were compared to alterations in the yellow areas of senescent leaves as well as mature summer leaves.

## 2. Results

**Figure 1:**
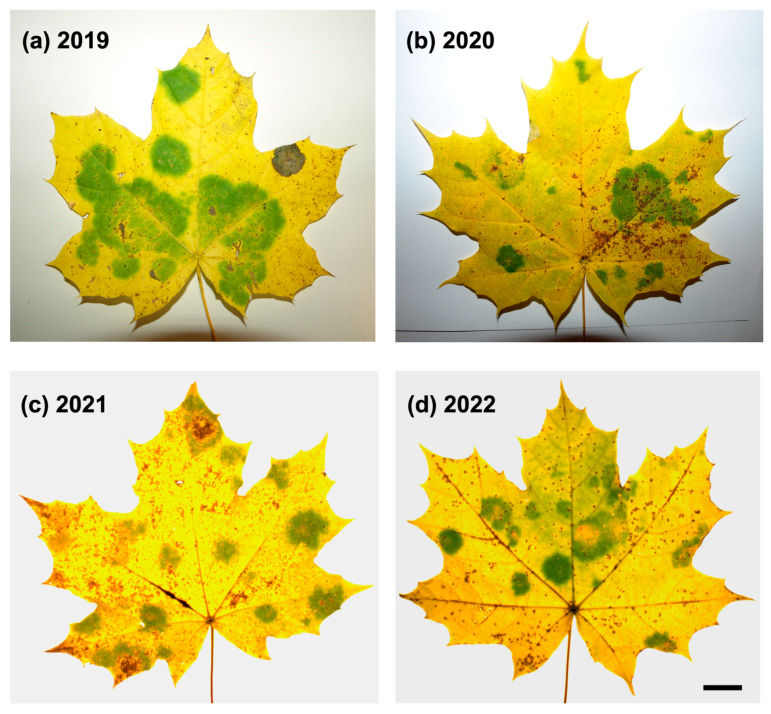
Representative images of *A. platanoides* senescent leaves with ‘green islands’ symptoms harvested in October/November in the years 2019–2022. (**d**) bar = 10 mm.

All analyzed leaves with ‘green island’ symptoms were collected from young Norway maple trees in the last week of October/the first decade of November in the years 2019–2022 (Figure 1). The summer leaves were collected from the same trees in June 2020 and 2021.

### 2.1. ‘Green Island’ Symptoms

**Figure A1:**
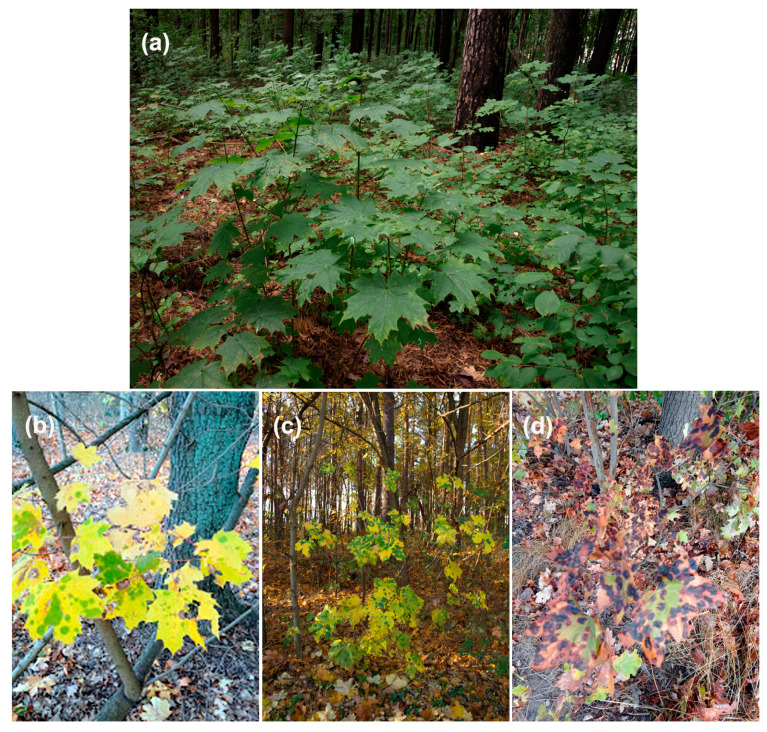
Young maple trees from the urban forest on the University of Bialystok campus (Bialystok, Poland). (**a**) Green leaves of Norway maple on 27 June 2021; (**b**) Norway maple senescent leaves with ‘green islands’ symptoms on 5 November 2022; (**c**) young field maple trees with ‘green islands’ symptoms on 9 November 2020; (**d**) Norway maple leaves with tar spot symptoms on 27 September 2024.

‘Green islands’ on the leaves of young Norway maples were first observed in late October 2019, when leaves began to yellow (Figure 1a). ‘Green islands’ are vividly green and mostly round, with various sizes from approximately 0.5 to 4.5 cm in diameter. Between one (relatively rarely) and ten ‘green islands’ per leaf were formed. These features differed ‘green islands’ from irregular patches of green tissue in senescent leaves. Most young and old Norway maple trees within the urban forest surrounding the University of Bialystok campus displayed ‘green island’ symptoms (Figure A1b).

Moreover, ‘green islands’ could also be observed on the leaves of another maple species, field maple (*Acer campestre* L.) (Figure A1c). Maples showing ‘green island’ symptoms could not be found in other locations of the urban forest in Bialystok. In the second half of November 2019, the fallen leaves around trees had still-evident ‘green islands’. In 2020–2022, ‘green islands’ were major symptoms of biotic stress in maples (Figure 1), apart from the abundant leaf-biting areas caused by insects.

The mean percentage of ‘green island’ areas covering a leaf lamina changed within research years, showing significantly higher values in 2019 and 2021 compared to 2020 and 2022 (Table 1). A one-way analysis of variance revealed that the percentage of the ‘green island’ area was significantly influenced by a year of collection (F = 609.158, *p* < 0.001).

**Figure A2:**
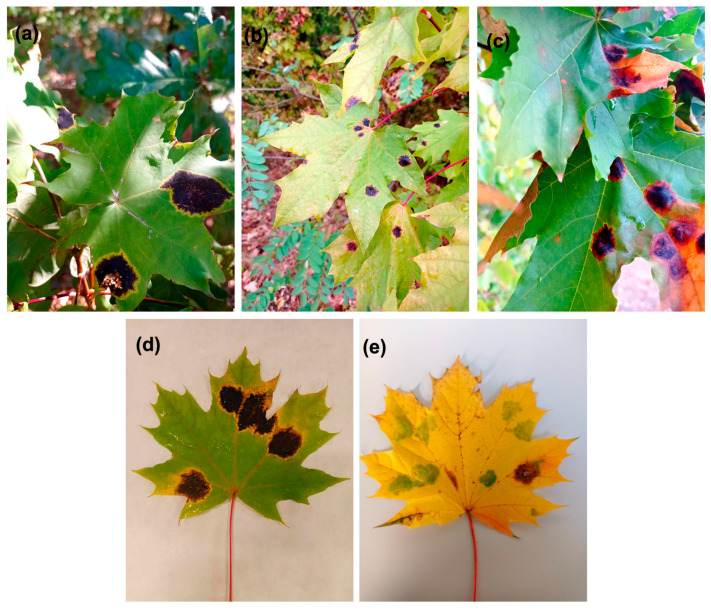
Tar spot symptoms on leaves of young Norway maple trees in the urban forest on the University of Bialystok campus (Bialystok, Poland). (**a**–**c**) Tar spots with yellow rings in late August 2023; (**d**) tar spots in September 2024; (**e**) ‘green island’ symptoms with a single tar spot in November 2024.

From 2022, in the first decade of September, the tar spot, caused by a pathogenic fungus of *Rhytisma* spp., occurred on only a few Norway maple leaves (Figure A1d). This disease started in the form of yellowing leaf spots in late August 2023, and the round black tar spots were surrounded by yellow rings in the middle of September (Figure A2a,d). Tar spot was a major disease observed in Norway maples in 2023. Only a few young trees showed ‘green island’ symptoms. However, tar spots together with ‘green islands’ could also be found on the same leaves. A similar correlation of ‘green islands’ and tar spot appearance was also observed in 2024 (Figure A2).

### 2.2. Non-Destructive Assessment of Physiological Parameters

Three variants of leaves were assessed in the non-destructive physiological analysis of chlorophyll, flavonoids, anthocyanins, and nitrogen balance index (NBI): green mature summer leaves; and two areas of autumn leaves, being ‘green islands’ and senescent yellow areas. The analyses were performed every year on leaves collected from 2019 to 2021.

The content of chlorophyll and flavonoids and indices of nitrogen balance (NBIs) in ‘green islands’ were significantly lower than in the summer leaves, but higher compared to the senescent yellow area of the autumn leaves (Table 2). These parameters were similar in the ‘green islands’ of senescent leaves harvested in 2019 and 2021 and differed significantly compared to 2020. In the case of the summer leaves and the yellow areas of senescent leaves, chlorophyll, flavonoids, and NBIs were at the same level in each examined year. However, anthocyanin content was significantly higher in yellow areas than in ‘green islands’, although, in the summer leaves, anthocyanins were undetectable (Table 2).

A two-way analysis of variance revealed that the content of chlorophyll, flavonoids, and anthocyanins were significantly influenced by a year of sampling (at least *p* < 0.005) and leaf variant (at least *p* < 0.005) (Supplementary Materials Table S1). Only the NBIs were not influenced by a year of sampling (F = 0.07, p = 0.8), although the NBIs showed significant dependence on a leaf variant (F = 5429.69, p < 0.001).

### 2.3. Chlorophyll and Carotenoid Contents

An analysis of chlorophyll and carotenoid content was performed in three variants of leaves: green mature summer leaves, and two areas of the autumn leaves: ‘green islands’ and senescent yellow areas. The leaves were collected in the summer and autumn 2020.

The content of chlorophyll *a*, *b*, and total chlorophyll differed significantly between leaf variants, showing the highest values for the summer leaves and 4–7 times lower values in the yellow areas of the autumn leaves (Table 3). In the case of ‘green islands’, the amounts of chlorophyll were only 1.3–1.4 times lower than in the summer leaves and approximately 3–4.5 higher than in the yellow areas. Surprisingly, the ratio of chlorophyll *a*:*b* was the same in the summer leaves and ‘green islands’, regardless of the lower content of chlorophylls in ‘green islands’. This parameter was significantly lower in the yellow areas of the autumn leaves than in the other two leaf variants (*p* < 0.001; Table 3).

Carotenoid content had almost the same value in the ‘green island’ and yellow areas of the autumn senescent leaves. In the summer leaves, there were significantly fewer carotenoids than in the autumn leaves (*p* < 0.001). The ratio of total chlorophyll:carotenoids was extremely high in the summer leaves (18.627 ± 8.63) and over three times lower in ‘green islands’. In the yellow areas of the autumn leaves, this parameter was significantly lower than in the other examined leaf variants (Table 3). One-way analysis of variance revealed that all calculated parameters were significantly influenced by a leaf variant (at least *p* < 0.001).

### 2.4. Analysis of Photosynthetic Parameters

A non-destructive analysis of chlorophyll fluorescence was performed on green mature summer leaves and two areas of the autumn leaves: the ‘green islands’ and senescent yellow areas collected in the summer and autumn of 2020.

In the ‘green island’ areas, the JIP test parameters took values intermediate between those for the green summer leaves and the yellow areas of the senescent leaves (Table 4). The maximum photochemical efficiency of the PSII (Fv/Fm) of ‘green islands’ on senescent leaves was slightly reduced compared to the summer leaves (10%) and more than two times higher than in the yellow areas. Despite this, a significant decrease in the PI abs value was revealed. A significant decrease in Fv/F0 was also observed, with a simultaneous increase in the values of parameters describing energy absorption by active reaction centers (ABS/RC, TR0/RC) and energy dissipation in non-photochemical form (DI0/RC) in green islands compared to summer leaves. However, in senescent leaves, the fluctuations of the above parameters were significantly greater than in the ‘green island’ areas (Table 4). The probability (t0) that a trapped exciton moved an electron into the electron transport chain beyond QA (ΨEo) was at a similar level in the ‘green island’ and yellow parts of the senescent leaf (more than half as low as in summer leaves). The efficiency with which an electron from the intersystem carriers moves to reduce end electron acceptors at the PSI acceptor side (at t0) increased in senescent leaves and reached the highest values in yellow areas; however, the quantum yield for reduction in end electron acceptors at the PSI acceptor side (ϕRo) was similar in all of the analyzed leaf tissues (Table 4).

### 2.5. Anatomy and Ultrastructure of Leaves

**Figure 2:**
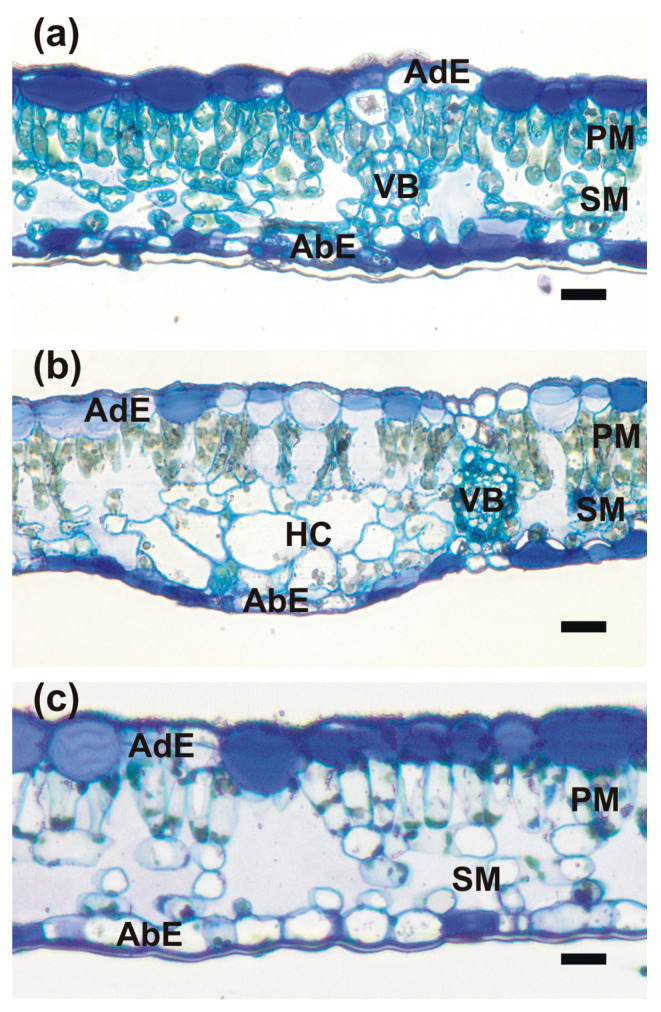
Light microscopy images of Toluidine Blue-stained sections taken from the following: (**a**) mature summer leaf; (**b**) ‘green island’ induced on autumn senescent leaf; (**c**) the yellow area of a senescent leaf. Abbreviations: AbE, abaxial epidermis; AdE, adaxial epidermis; HC, hypertrophied cells; PM, palisade mesophyll; SM, spongy mesophyll; VB, vascular bundle. Scale bars: 20 μm.

**Figure 3:**
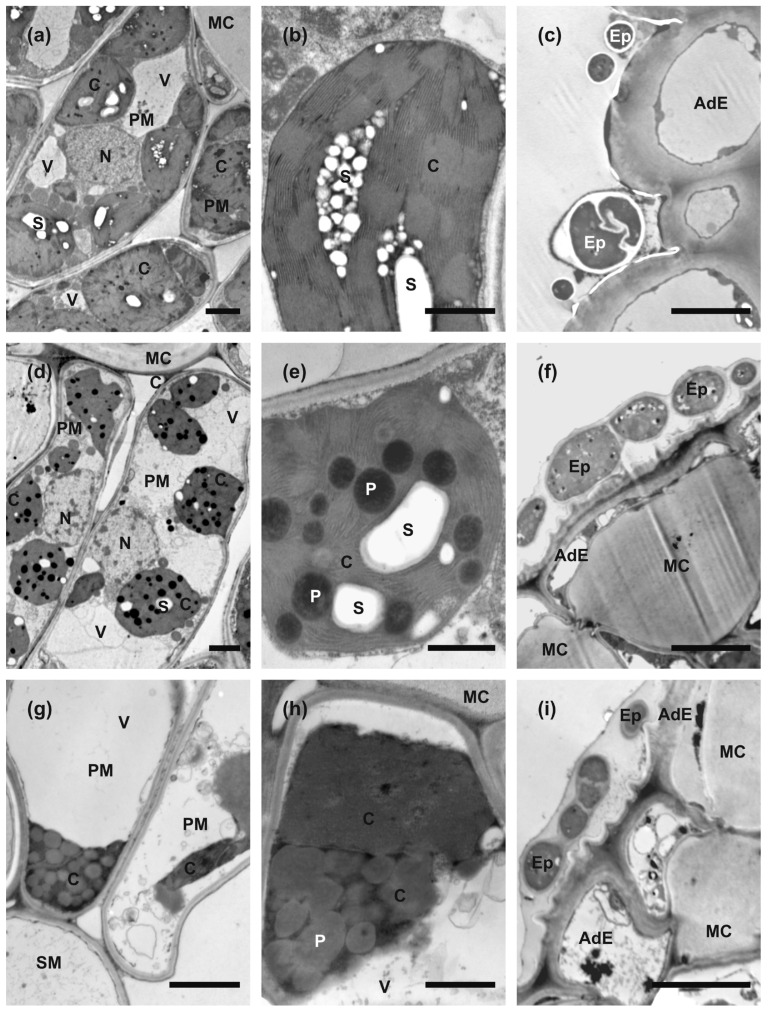
Transmission electron microscopy images of sections taken from summer green leaf (**a**–**c**), ‘green island’ induced on autumn senescent leaf (**d**–**f**), and the yellow area of a senescent leaf (**g**–**i**). (**a**,**d**,**g**) Overview of palisade mesophyll cell ultrastructure. (**b**,**e**,**h**) Overview of chloroplast’s ultrastructure. (**c**,**f**,**i**) Epiphytes located on the surface of adaxial epidermis. Abbreviations: AdE, adaxial epidermis; C, chloroplast; Ep, epiphyte; MC, mucilaginous cell wall; N, nucleus; P, plastoglobuli; PM, palisade mesophyll cell; S, starch grain; SM, spongy mesophyll cell; V, vacuole. Scale bars: 5 μm (**c**,**f**,**i**); 2 μm (**a**,**d**,**g**), and 1 μm (**b**,**e**,**h**).

The anatomy of leaf blades was clearly recognizable in all collected samples (Figure 2). The leaves were covered by single-cell layers of adaxial and abaxial epidermis surrounding the mesophyll, which was composed of a single tier of relatively short palisade mesophyll cells and 3–4 tiers of loosely arranged spongy mesophyll cells. The extensive intercellular spaces were present in the latter. The uncommon feature of epidermal cells was the strong thickening of inner cell walls which were infiltrated with mucilage, and thus were strongly stainable with Toluidine Blue (Figure 2 and Figure 3f,i). The mucilaginous cell walls were present in most of the adaxial epidermis cells, except the cells above the vascular bundles, which retained non-modified call walls. In the abaxial epidermis, their development was more random, but most of the epidermises had mucilaginous cell walls (Figure 2). Cross-sections of the ‘green island’ regions show a slightly less compact arrangement of palisade and spongy parenchyma cells in comparison to the mature summer leaves (Figure 2b). The outlines of mesophyll cells were strongly wavy. Green-stained chloroplasts were still clearly recognizable and located along the cell walls. Large intercellular spaces were formed even between palisade mesophyll cells. Additionally, groups of strongly hypertrophied cells were often found next to the vascular bundles. The anatomical organization was still well-preserved in the yellow areas of the leaf blade, but mesophyll cells seemed to be almost empty or contained single patches of strongly stained protoplast remnants (Figure 2c). The vascular bundles in ‘green island’ regions contained well-preserved phloem and xylem parenchyma cells, whereas these cells were degraded in yellow areas.

Ultrastructural examinations of control summer leaves indicated that palisade mesophyll cells protoplasts contain numerous large chloroplasts, nuclei with an electron-dense nucleoplasm, and relatively small vacuoles (Figure 3a). Spongy mesophyll cells contained fewer and smaller chloroplasts and large central vacuoles. Chloroplasts were generally round in outlines on sections. Their stroma was strongly electron dense; the thylakoid system was arranged in extensive grana, and large starch grains were formed. Almost no plastoglobuli were observed (Figure 3b). In contrast, palisade mesophyll cells in ‘green island’ samples contained large central vacuoles, and chloroplasts were located in cytoplasm along the cell walls (Figure 3d). Additionally, nucleoplasm was also more electron-translucent (Figure 3d). The chloroplasts were also smaller and had more irregular outlines than chloroplasts in mature summer leaves. Moreover, chloroplasts had electron-dense stroma and a well-developed system of thylakoids, but the grana were hardly discernible (Figure 3e). In samples collected from the yellow areas of autumn leaves, mesophyll cells contained only remnants of degraded protoplasts (Figure 3g). Chloroplasts could be recognized only as strongly osmiophilic spots with numerous plastoglobuli inside (Figure 3h), and they contained smaller and fewer starch grains in contrast to mature green leaves.

Spores or hyphae of epiphytes were attached to the surface of adaxial epidermis in all types of samples. In mature summer leaves, they were relatively few, and only sections of single cells were observed (Figure 3c). However, in ‘green island’ and yellow areas, they appeared frequently as sections of multicellular hyphae embedded in a mucilaginous sheath (Figure 3f,i). No section showing the formation of invasion hyphae or hyphae inside or between mesophyll cells was found. Thus, the nature and function of these epiphytes could not be established clearly.

## 3. Discussion

Green spots on senescent leaves, called ‘green islands’, are a seasonal phenomenon associated with autumn and may be caused by pathogens attacking a weakened plant and senescent tissues enhance their visibility. They are most often related to the colonization of plant tissues with pathogenic fungi that are obligate biotrophs or hemibiotrophs at their biotrophic stage [11,22]. In the case of biotrophic pathogens, the inhibition of naturally occurring or induced senescence is beneficial for these pathogens, as they rely on living host cells. However, it is more challenging to explain the formation of ‘green islands’ in the case of necrotrophs, as they require dying or dead cells to feed on [21].

‘Green islands’ are leaf lamina fragments in which there is an increased concentration of cytokinins and organic nutrients [23]. As plant hormones, cytokinins are involved in various biological processes, including the inhibition of plant cell senescence. They are also responsible for regulation and maintaining an appropriate level of chlorophyll, thus increasing the mobilization of nutrients [24]. Environmental factors, such as temperature or humidity, may also affect ‘green island’ development, increasing the intensity of plant–pathogen interactions [25]. ‘Green islands’ on Norway and field maple leaves could be observed in the following years across the temperate climate zone of Europe, which has four clear seasons [15]. Moreover, they were also described on apple leaves, induced by leaf-mining insects (*Phyllonorycter blancardella*), and the presence of the endosymbiotic bacteria *Wolbachia* enhanced their appearance by the modulation of host physiology [26].

This work studied the appearance of ‘green islands’ on Norway maple trees in eastern Poland during 6 years, 2019–2024 (Figure 1 and Figure A1). However, very dry winters almost without snow or rain and warm summers (hydrological drought) probably led to the disappearance of ‘green islands’ on Norway maples in 2023 and 2024 [27,28]. Instead, tar spot, another fungus-induced disease of maples, occurred (Figure A1 and Figure A2). However, how drought and elevated temperature may influence ‘green island’ formation needs to be investigated.

Although, ‘green islands’ were vividly greenish even on fallen senescent leaves of Norway maples in the late autumn (November), we decided to investigate whether these green spots were still alive and photosynthetically active. Photosynthesis is the primary process of the plant metabolism, which, in addition to biotic and abiotic environmental factors, is influenced by the structure of assimilation organs (mainly leaves). Significant differences in the efficiency of photochemical processes in individual leaf parts has already been well understood, as well as influence of the efficiency of the photosynthesis light phase on other ontogenetic processes occurring in the leaf, e.g., those related to generative development [29]. Thus, non-invasive estimations of chlorophyll, defense-related metabolites (flavonoids), and the NBI index of plant health status indicated that ‘green island’ tissues were still alive. However, the mean values of those parameters were significantly lower than in summer healthy leaves (Table 2 and Table 3). Chlorophyll content is very often negatively affected by both biotrophic and necrotrophic pathogens and depends on water status, and enhanced hydrological drought (which fluctuated between years [27,28]) may affect ‘green island’ formation and chlorophyll maintenance [21,30]. In this study, the highest amounts of chlorophylls (total Chl, Chl *a* and *b*) were observed in summer leaves, significantly lower in ‘green islands’, and the lowest chlorophyll contents were noticed in yellow area of senescent leaves (Table 3). Such a pattern of chlorophyll contents could be observed, for, e.g., in yellow areas and dark ‘green islands’ (DGIs) induced by Cucumber Mosaic Virus in *Nicotiana tabacum* [31]. A negative correlation between the ‘green island’ area and chlorophyll content over the years could be related to the fluctuation in carotenoid content, which was relatively high in ‘green islands’ (similar to yellow areas), but we investigated it only in one year, or else unbalanced nitrogen content as NBI also showed negative correlation to green island areas (and chlorophyll contains nitrogen). One more possible explanation for these negative correlations could be a fluctuating microbiome composition over the years, but it needs to be investigated. In general, the biosynthesis of flavonoids in many cases increases during a pathogen infection as a part of the activity of a host defense system [32,33]. So, it is unsurprising that flavonoid contents were significantly higher in senescent leaves both in ‘green islands’ and yellow areas. Moreover, the mean values of flavonoids were the least fluctuating parameters over the years in all investigated leaf variants. As expected, anthocyanins were undetectable in summer leaves compared to the high amounts of anthocyanins in the yellow areas of senescent leaves. Also, significantly increased amounts of anthocyanins were detected in ‘green islands’ (Table 2). All leaf variants had similar mean anthocyanin contents in 2019 and 2020. However, they significantly increased (twice as much) in 2021 both in the ‘green islands’ and yellow areas of senescent leaves, which is difficult to explain based on the other obtained results. Anthocyanins are often associated with low chlorophyll levels; thus, they appear in abundance in senescent tissues [34]. Moreover, they also play a photoprotective role during senescence to cope with excess light similarly as carotenoids [35,36]. It has to be emphasized that such physiological analyses are rarely performed during ‘green island’ appearances, so it is difficult to compare our results to other research concerning ‘green island’ development.

Moreover, a detailed analysis of photosynthetic parameters has been performed (Table 4). The description of light energy transfer within PSII is possible by using the analysis of chlorophyll *a* fluorescence kinetics parameters. The light-harvesting complex of photosystem II absorbs light reaching the leaf. Then the electrons are transported to the central part of the antenna and chlorophyll molecules constituting the reaction centers (RC). This results in charge separation across the membrane and splitting of water molecules into molecular oxygen protons and electrons on the donor side of PSII. Furthermore, electron transport from PSII involves their transfer to QA and QB via b6f, and plastocyanin to PSI, where a second charge separation and further reduction in ferredoxin and NADP+ occur [37]. Thanks to the advanced methodology, the efficiency of the above processes can be effectively analyzed using the JIP test, which allows for the early detection of disturbances in the light phase of photosynthesis [38,39].

The maximum photosynthetic quantum efficiency (Fv/Fm) in ‘green islands’ was slightly reduced (10%) compared to summer leaves (Table 4). Related to ‘green islands’ resulting from the impact of biotrophic fungal pathogens, this decrease was small (in barley leaves during *Blumeria graminis* infection, a decrease in the quantum efficiency was 47% in the ‘green island’ tissues) [11]. At the same time, Fv/Fm remained significantly higher in the ‘green islands’ compared to the yellow areas of the senescing leaf, which is consistent with previous observations made on plants of other species [40]. Despite this, we observed a significant decrease in Fv/F0 (compared to summer leaves), which indicated the disturbance of activity of the water-splitting complex on the donor side of PSII or the damage of thylakoid structure [38,41]. However, the literature provides conflicting information about whether chloroplasts in ‘green islands’ remain intact and photosynthetically active. The decrease in the PI abs value, with a simultaneous increase in TR0/RC and DI0/RC, suggests a decrease in the number of active reaction centers and decreased efficiency of electron transport beyond QA, with a simultaneous high degree of electron trapping in the PSII antenna (increase in the size of the PSII antenna) and dissipation of a significant amount of absorbed energy as heat [42]. This phenomenon is probably related to the delayed distribution of antennal chlorophyll in the ‘green island’ tissues (Table 4).

The only research on ‘green islands’ on maples revealed that Norway maple leaves were mainly infected by endophytic bacteria from the *Gramproteobacteria* family, and the most numerous endophytic fungi belonged to *Dothideomycetes* and *Leotiomycetes* [15]. In this study, only the spores and hyphae of epiphytic fungi were detected on all leaf variants during microscopic analyses. Moreover, they were present in abundance on both the yellow areas and ‘green islands’ of senescent leaves (Figure 3). However, no fungal cells were observed inside mesophyll.

An ultrastructural analysis of the leaves showed that cells located in the unevenly distributed ‘green islands’ on senescent leaves differed from those building a healthy summer leaf. ‘Green islands’ have more developed mesophylls than in the yellow area of the senescent leaf. So, ‘green island’ development leads to an extension of the photosynthesis process, which is beneficial for biotrophic pathogenic and non-pathogenic epiphytes and endophytes to be active longer in autumn season [43,44]. However, extended photosynthesis can also lead to a deficiency of nutrients, such as nitrogen, resulting in a weakening of the plant, which may have difficulties to survive the winter, the consequences of which will be visible in spring when the trees re-green [45,46]. The most conspicuous features of cells in ‘green island’ mesophylls were smaller chloroplasts with numerous plastoglobuli compared to summer leaves (Figure 3). This phenomenon was also observed in *Triticum aestivum* ‘green island’ cells during infection with *Puccinia striiformis* [40] and in *Brassica juncea* ‘green islands’ infected with *A. brassicicola* [21]. The abundance of plastoglobuli could be a response to extensive lipid peroxidation that occurred in ‘green island’ cells, which was detected as a significant increase in malondialdehyde (MDA) content in comparison to their amounts in summer leaves and the yellow areas of senescent leaves (Figure S1). Elevated MDA content is a marker of oxidative stress, and it is often observed in host cells during pathogen infection and abiotic stress [47].

Further research on ‘green island’ origin and functioning will allow for a more detailed understanding of the causes of this phenomenon and its role in plant–pathogen interactions.

## 4. Materials and Methods

### 4.1. Plant Material

All experiments were performed on Norway maple (*Acer platanoides* L.) leaves plucked from young 6–8-year-old trees (Figure A1). The trees were localized in the urban forest surrounding the University of Bialystok campus (53°06′56″ N 23°09′54″ E) in Bialystok, a city in eastern Poland. The young summer leaves were collected from Norway maples on 27 June 2020 and 7 June 2021, whereas the senescent leaves showing ‘green island’ symptoms were collected in the first week of November in the years 2019–2022. The leaves were torn from young maples (fallen leaves were not collected) and carefully selected to avoid collecting leaves with blurred, irregular green spots, which could result from a delayed aging process. In each experiment, three variants of leaf areas were analyzed: summer leaves and two areas within senescent leaves, yellow and ‘green island’ areas. The Norway maple trees were also observed for ‘green island’ symptoms in the following years, 2023 and 2024. However, due to the small number of leaves showing ‘green islands’, the leaves were not subjected to analysis.

### 4.2. Non-Destructive Evaluation of Physiological Parameters

Each year, beginning from the autumn of 2019 until November 2022, the ‘green island’ areas were measured in senescent leaves using a WinDIAS Leaf Image Analysis System (Delta-T Devices, Cambridge, UK) and are expressed as a percentage of the whole leaf surface. Each year, between 9 and 30 leaves per variant were analyzed.

The same leaves plus the additional number of those harvested on the other day of November were subjected to non-destructive measurements of physiological parameters. Chlorophyll content in the mesophyll, anthocyanin and flavonoid content in the leaf epidermis, and the Nitrogen Balance Index (NBI; the ratio of chlorophylls and flavonoids) were measured with a Dualex Optical Leaf-Clip Sensor (ForceA, Orsay, France). This method is based on leaf transmittance and chlorophyll fluorescence [48]. The content of chlorophylls is expressed in μg cm^−2^, whereas anthocyanins, flavonoids, and NBI have no units [49]. The measurements were also performed on 25–65 summer leaves. A detailed statistical analysis of these parameters is available in Supplementary Materials Table S1.

### 4.3. Chlorophyll and Carotenoid Content

The 150 mg samples from the summer and autumn leaves were collected on 27 June and 10 November 2020, respectively, and stored at −80 °C until used. Chlorophylls and carotenoids were extracted in 100% methanol (HPLC grade, Avantor Performance Materials, Gliwice, Poland), as described by Macioszek et al. [32], analyzed using a Hitachi U-5100 spectrophotometer (Hitachi Ltd., Tokyo, Japan), and calculated according to Wellburn [50]. Eight summer and eight autumn leaf samples were used for analyses, with four technical replicates.

### 4.4. Analysis of Photosynthesis

For the chlorophyll fluorescence analyses, from 21 to 58 detached summer and senescent leaves were immediately dark-adapted for 20 min using leaf clips. Then, dark-adapted areas were subjected to measurements conducted with a Pocket PEA chlorophyll fluorimeter (Hansatech Instruments Ltd., Norfolk, UK) equipped with a high-intensity focus LED of 3500 µmol m^−2^ s^−1^ photon flux with a peak wavelength of 627 nm at the sample surface according to the manufacturer’s built-in protocol. Leaves for analyzes were collected in June and November 2020.

### 4.5. Microscopic Analysis

The leaves for light and transmission electron microscopy investigation were collected on 7 June and 10 November 2021. The samples were dissected from green summer leaves and two areas of autumn leaves: yellow and the center of the ‘green islands’. They were collected from at least three different trees. The dissected specimens were immediately transferred into a modified Karnovsky fixative and processed for light and transmission electron microscopy and sectioning, as described by Crespo et al. [51].

### 4.6. Statistical Analysis

Each parameter’s means and standard deviations (SD) were calculated using MS Office Excel 2021 software. An analysis of variance (ANOVA) and post hoc Duncan’s test (*p* < 0.05) of all the data obtained in this work were performed using STATISTICA v.13.3 (Tibco Software Inc., StatSoft, Krakow, Poland).

The figures were composed using Adobe Photoshop v. 6.0 and Corel Software v. 11.

## 5. Conclusions

‘Green islands’ have been described in limited but variable pathosystems, mainly affecting crop plants such as maize, barley, and tobacco [16,18,20]. However, naturally occurring ‘green islands’ were observed only in a few tree species, such as maples and apples [15,26]. Pathogens and insects inducing ‘green islands’ also have different life cycles and feeding strategies. Therefore, the structural, functional, and environmental factors of ‘green islands’ require further research, as do the biotic and abiotic factors influencing their appearance. The physiological, metabolic, and genetic aspects of ‘green islands’ also should be investigated.

The potential usefulness of ‘green islands’ cannot be overestimated. ‘Green islands’ can indicate specific pathogen infection; virus-induced ‘green islands’ look different from those on maple leaves. Moreover, they can be an interesting model for studying how pathogens manipulate host metabolism (e.g., this study) or for observing localized immune responses similarly to hypersensitive responses. A more far-reaching use of ‘green islands’ will be the identification of the genetic traits associated with resistance to pathogens that induce ‘green islands’ to support breeding programs for disease-resistant varieties. ‘Green islands’ constitute a research challenge due to their low impact and economic significance on tree leaves and their visibility being mostly in autumn.

## Figures and Tables

**Table 1:** Mean percentages of ‘green island’ area in *A. platanoides* leaves (n = 9–30) evaluated each year in November from 2019 to 2022. Data were gained using the WinDIAS system. Statistical differences between the means were labeled with different letters according to a post hoc Duncan’s test (*p* < 0.05).

	2019	2020	2021	2022
Percentage of ‘green’ island area	23.99 a	15.60 b	26.17 a	19.49 ab
SD	9.62	4.61	6.43	4.42

**Table 2:** Non-destructive measurements of chlorophyll (Chl), flavonoids (Flav), anthocyanins (Anth), and nitrogen balance index (NBI) in *A. platanoides* leaves. Data were gained using a Dualex sensor. The means (n = 25–65) ± SD were obtained from senescent leaves harvested each year in November from 2019 to 2021 and from summer leaves harvested between June 2020 and 2021. Different letters within each year indicate significant differences between leaf variants according to a post hoc Duncan’s test (*p* < 0.05).

Year	Leaf Variant	Chl (µg/cm^2^)	Flav Index	Anth Index	NBI
2019	‘Green island’ Yellow area	11.34 ± 5.40 a	1.06 ± 0.22 a	0.27 ± 0.07 a	11.09 ± 5.60 a
		3.35 ± 1.96 b	1.25 ± 0.20 b	0.38 ± 0.05 b	2.74 ± 1.68 b
2020	Summer leaves	35.91 ± 2.63 c	0.90 ± 0.06 c	0.00	40.10 ± 3.77 c
	‘Green island’ Yellow area	15.85 ± 5.25 d	1.02 ± 0.22 a	0.24 ± 0.05 a	16.50 ± 7.37 d
		2.47 ± 1.33 b	1.24 ± 0.16 b	0.38 ± 0.05 b	2.02 ± 1.10 b
2021	Summer leaves	34.15 ± 3.62 c	0.80 ± 0.14 c	0.00	43.99 ± 9.71 e
	‘Green island’ Yellow area	12.75 ± 2.29 a	0.96 ± 0.16 a	0.63 ± 0.05 c	13.38 ± 2.27 a
		1.99 ± 0.97 b	1.06 ± 0.21 a	0.88 ± 0.06 d	1.89 ± 0.78 b

**Table 3:** Chlorophyll (Chl) and carotenoid (Car) content in *A. platanoides* leaves determined spectrophotometrically. The means (n = 8) ± SD were obtained from senescent leaves harvested in November 2020 and summer leaves harvested in June 2020. Different letters within each parameter indicate significant differences between leaf variants according to a post hoc Duncan’s test (*p* < 0.05).

Parameters	Summer Leaves	Senescent Leaves	
(μg mg^−1^ F.W.)		‘Green Islands’	Yellow Area
Chl*a*	1.933 ± 0.10 a	1.395 ± 0.27 b	0.268 ± 0.09 c
Chl*b*	1.156 ± 0.19 a	0.814 ± 0.14 b	0.272 ± 0.08 c
Chl*a:b*	1.709 ± 0.27 a	1.709 ± 0.08 a	1.00 ± 0.22 b
Total Chl	3.09 ± 0.24 a	2.209 ± 0.42 b	0.540 ± 0.15 c
Car	0.190 ± 0.66 a	0.400 ± 0.06 b	0.431 ± 0.07 b
Total Chl:Car	18.627 ± 8.63 a	5.636 ± 1.40 b	1.201 ± 0.37 b

**Table 4:** Parameters of chlorophyll fluorescence in *A. platanoides* leaves. The means (n = 21–58) ± SD were obtained from summer leaves in June 2020 and autumn senescent leaves in November 2020. Data were gained using a PocketPEA fluorimeter. Different letters indicate significant differences between leaf variants according to a post hoc Duncan’s test (*p* < 0.05).

Parameters	Summer Leaves	Senescent Leaves	
		‘Green Islands’	Yellow Area
Measured parameters and basic JIP-test parameters			
Fo	866.7 ± 143.78 a	397.1 ± 125.22 b	561.74 ± 226.7 c
Fm	34,039 ± 1447 a	27,345 ± 5723 b	4452 ± 2818 c
Fv	26,032 ± 1232 a	18,997 ± 4913 b	1702 ± 1453 c
Fv/Fm	0.765 ± 0.012 a	0.688 ± 0.07 b	0.340 ± 0.128 c
Fv/Fo	3.261 ± 0.215 a	2.349 ± 0.641 b	0.581 ± 0.374 c
Vj	0.380 ± 0.032 a	0.733 ± 0.032 b	0.730 ± 0.102 b
Vi	0.929 ± 0.013 a	0.919 ± 0.023 a	0.827 ± 0.053 b
PI abs	4.268 ± 1.065 a	0.283 ± 0.151 b	0.029 ± 0.031 c
Specific energy fluxes expressed per active RC of PSII			
ABS/RC	1.294 ± 0.125 a	3.500 ± 0.875 b	10.392 ± 5.032 c
DIo/RC	0.306 ± 0.043 a	1.151 ± 0.623 b	7.389 ± 5.038 c
TRo/RC	0.988 ± 0.084 a	2.349 ± 0.291 b	3.003 ± 0.286 c
ETo/RC	0.611 ± 0.039 a	0.621 ± 0.067 a	0.813 ± 0.315 b
REo/RC	0.070 ± 0.012 a	0.194 ± 0.075 b	0.523 ± 0.167 c
Quantum yields parameters			
ϕ(Po)	0.765 ± 0.012 a	0.688 ± 0.074 b	0.340 ± 0.128 c
ψ(Eo)	0.620 ± 0.032 a	0.267 ± 0.032 b	0.270 ± 0.102 b
ϕ(Eo)	0.474 ± 0.029 a	0.185 ± 0.036 b	0.088 ± 0.041 c
Δ(Ro)	0.114 ± 0.019 a	0.308 ± 0.107 b	0.668 ± 0.181 c
ϕ(Ro)	0.054 ± 0.010 a	0.054 ± 0.010 a	0.056 ± 0.02 a

## Data Availability

Data are contained within the article or Supplementary Materials.

## Supplementary Materials

The following supporting information can be downloaded at: https://www.mdpi.com/article/10.3390/plants14060909/s1, Table S1: Two-way ANOVA of non-destructive parameters: chlorophyll, flavonoids, anthocyanins, and nitrogen balance index (NBI) in summer and autumn Norway maple leaves; Figure S1: Content of malondialdehyde (MDA) in summer and senescent leaves of Norway maple collected in June and November 2020.

## Appendix A

An abundant appearance of ‘green islands’ on Norway maple leaves was observed in the small area of the urban forest surrounding the University of Bialystok campus in Bialystok, eastern Poland (Figure A1). Tar spot, a fungal disease, could be observed on Norway maple leaves since 2022, and it became the major disease of Norway maple since 2023. Last year, only a few young trees showed symptoms of ‘green islands’, sometimes in combination with tar spots (Figure A2).
