# Supplementary material for: Phenological, Physiological, and Ultrastructural Analyses of ‘Green Islands’ on Senescent Leaves of Norway Maple (Acer platanoides L.)
Source: Plants (Basel). 2025 Mar 14;14(6):909. doi: 10.3390/plants14060909 (PMC11945391; doi:10.3390/plants14060909)
Supplement: Supplementary file 1 [file plants-14-00909-s001.zip › plants-3488110-supplementary.pdf]

Table S1. Two-way ANOVA of non-destructive parameters: chlorophyll, flavonoids, anthocyanins, and nitrogen balance index (NBI) in summer and autumn Norway maple leaves

	Source of variation	df	SS	MS	F	*p*
**Chl**	Year (Y)	1	108.39	108.39	7.67	**
	Leaf variant (LV)	1	4762.66	4762.66	337.25	***
	YxLV	3	598.96	199.65	14.14	***
	Error	462	6524.29	14.12		
**Flav**	Year (Y)	1	0.41	0.41	11.25	***
	Leaf variant (LV)	1	1.17	1.17	31.89	***
	YxLV	3	0.08	0.03	0.77	ns
	Error	459	16.80	0.04		
**Anth**	Year (Y)	1	3.13	3.13	689.20	**
	Leaf variant (LV)	1	1.24	1.24	271.81	**
	YxLV	3	1.90	0.63	139.20	**
	Error	446	2.03	0.004		
**NBI**	Year (Y)	1	1.57	1.57	0.07	ns
	Leaf variant (LV)	1	5429.69	5429.69	238.78	***
	YxLV	3	1040.94	346.98	15.26	***
	Error	459	10437.17	22.74		

Abbreviations: df – degrees of freedom; F – Fisher's F-test statistic; MS – mean sum of squares; SS – sum of squares. Levels of significance were: ** $p < 0.005$; *** $p < 0.001$; ns – non-significant.

Measurements of malondialdehyde (MDA)

Samples of 0.150 g of leaves tissue were homogenized in 2 ml of 5% (w/v) trichloroacetic acid (TCA; POCH Chemicals Co., Poland) solution containing 10 mM l^{-1} EDTA (Sigma, USA). Malondialdehyde (MDA) content was measured according to Heath and Packer [1968]. The supernatant was mixed with thiobarbituric acid (TBA; Sigma, USA) to determine the malondialdehyde content, which is a marker of lipid peroxidation. MDA levels were calculated as the difference of absorbance A532–A600 and shown as nM g^{-1} FW.

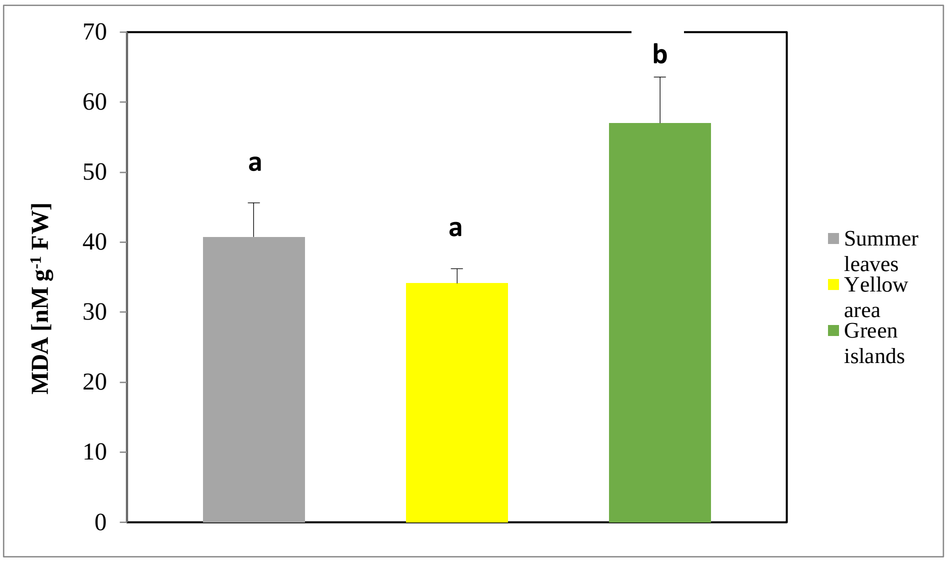

Figure S1. Content of malondialdehyde (MDA) in summer and senescent leaves of Norway maple collected in June and November 2020. Different letters indicate significant differences between leaf variants according to a post-hoc Duncan`s test ($p < 0.05$).

Heath, R.L.; Packer, L. Photoperoxidation in isolated chloroplasts. I. Kinetics and stoichiometry of fatty acid peroxidation. *Arch Biochem Biophys.* **1968**, *125*, 189–198. 10.1016/0003-9861(68)90654-1
